# Real-Time Neuropsychological Testing (RTNT) and Music Listening during Glioblastoma Excision in Awake Surgery: A Case Report

**DOI:** 10.3390/jcm12186086

**Published:** 2023-09-20

**Authors:** Grazia D’Onofrio, Nadia Icolaro, Elena Fazzari, Domenico Catapano, Antonello Curcio, Antonio Izzi, Aldo Manuali, Giuliano Bisceglia, Angelo Tancredi, Vincenzo Marchello, Andreaserena Recchia, Maria Pia Tonti, Luca Pazienza, Vincenzo Carotenuto, Costanzo De Bonis, Luciano Savarese, Alfredo Del Gaudio, Leonardo Pio Gorgoglione

**Affiliations:** 1 Clinical Psychology Service, Health Department, Fondazione IRCCS Casa Sollievo della Sofferenza, San Giovanni Rotondo, 71013 Foggia, Italy; 2 Complex Unit of Neurosurgery, Fondazione IRCCS Casa Sollievo della Sofferenza, San Giovanni Rotondo, 71013 Foggia, Italy; nadia.icolaro@gmail.com (N.I.); elena.fazzari87@gmail.com (E.F.); domenicocatapano1@gmail.com (D.C.); kino.23@virgilio.it (V.C.); costanzo.debonis@operapadrepio.it (C.D.B.); l.savarese@operapadrepio.it (L.S.); l.gorgoglione@operapadrepio.it (L.P.G.); 3 Division of Neurosurgery, BIOMORF Department, University of Messina, 98122 Messina, Italy; antonello.curcio@gmail.com; 4 Complex Unit of Anesthesia-2, Fondazione IRCCS Casa Sollievo della Sofferenza, San Giovanni Rotondo, 71013 Foggia, Italy; antonioizzi1201@gmail.com (A.I.); a.manuali@operapadrepio.it (A.M.); giulianobisceglia@live.it (G.B.); angelotancredi2@virgilio.it (A.T.); vincenzomarchello@libero.it (V.M.); a.recchia@operapadrepio.it (A.R.); mariapiatonti@alice.it (M.P.T.); freddydelgaudio@libero.it (A.D.G.); 5 Complex Unit of Radiology, Fondazione IRCCS Casa Sollievo della Sofferenza, San Giovanni Rotondo, 71013 Foggia, Italy; lucapazienza@libero.it

**Keywords:** awake surgery, real-time neuropsychological testing, music, glioblastoma, cognitive impairment, depression, anxiety

## Abstract

**Simple Summary:**

In a case study, real-time neuropsychological testing (RTNT) and music listening were applied for resections in the left temporal–parietal lobe during awake surgery (AS). The preoperative, intraoperative, and postoperative neuropsychological evaluation of patients with brain tumors and those treated neurosurgically has become part of clinical protocols. It allows for the evaluation of the presence of any neuropsychological deficits and their severity, provides reliable indications regarding the patients’ tolerability of an intervention in AS, and examines the cognitive and emotional motivational status of patients in the postoperative phase, thereby providing indications of the rehabilitation treatment and quality-of-life level. Moreover, we demonstrated that before/during AS and after music listening, the patient reported a decrease in depression and anxiety, in addition to an improvement in all the collected cognitive parameters. In conclusion, RTNT (also integrated with music listening) maximizes the surgical resection of lesions and minimizes the risks of post-operative neuropsychological and neurological sequelae through improving the quality of life of patients.

**Abstract:**

In this case report, real-time neuropsychological testing (RTNT) and music listening were applied for resections in the left temporal–parietal lobe during awake surgery (AS). The case is based on a 66-year-old with glioblastoma and alterations in expressive language and memory deficit. Neuropsychological assessment was run at baseline (2–3 days before surgery), discharge from hospital (2–3 days after surgery), and follow-up (1 month and 3 months). RTNT was started before beginning the anesthetic approach (T0) and during tumor excision (T1 and T2). At T0, T1, and T2 (before performing neuropsychological tests), music listening was applied. Before AS and after music listening, the patient reported a decrease in depression and anxiety. During AS, an improvement was shown in all cognitive parameters collected at T0, T1, and T2. After the excision and music listening, the patient reported a further decrease in depression and anxiety. Three days post surgery, and at follow-ups of one month and three months, the patient reported a further improvement in cognitive aspects, the absence of depression, and a reduction in anxiety symptoms. In conclusion, RTNT has been useful in detecting cognitive function levels during tumor excision. Music listening during AS decreased the patient’s anxiety and depression symptoms.

## 1. Introduction

Glioblastomas (GBMs) [1] are highly malignant tumors categorized as adult-type diffuse gliomas by the WHO in 2021. The new WHO classification system for brain tumors, published in 2021, has modified the nomenclature, creating a new family called “Adult-type diffuse gliomas”, which includes the following: astrocytoma, IDH-mutant; oligodendroglioma, IDH-mutant, and 1p/19q-codeleted; glioblastoma, IDH-wildtype. For simplicity, the authors refer to this family as “GBM”. GBMs are the most common adult brain tumors, with an annual incidence of approximately over 4/100,000 [1,2,3]. They occur predominantly between the ages of 45 and 64 years [4]. The tumor often has a rapidly progressive course (around 2–3 months). The median overall survival time is around 14.6 months, with a 5-year survival rate of only 7.2% [5,6]. The neurological signs are nonspecific as they are secondary to intracranial hypertension and/or behavioral changes and/or focal neurologic deficits [7].

The most frequent deficits detected in patients with GBM, through a pre- and post-operative cognitive assessment, were identified in executive functions, working memory, and attention [8,9,10,11,12]. The drastic worsening of quality of life experienced by patients who present a worsening in daily performance due to cognitive deficits is described in the literature, and it showed that cognitive rehabilitation could significantly improve performance [11,12,13]. Brain tumor patients often present with symptoms related to profound fatigue that prevents them from being active and reduces their social participation [12]. Therefore, the maintenance of language and cognition is essential in GBM surgery because they are fundamental features of daily life performance [13]. In a study, it was confirmed via a pre- and post-operative neuropsychological evaluation that awake surgery (AS) is associated with good cognitive and linguistic clinical outcomes in malignant tumors [14].

Moreover, detailed information provided about the cognitive status of patients during AS using a neuropsychological monitoring technique called real-time neuropsychological testing (RTNT) is considered necessary [14]. RTNT includes testing protocols based on the area where the surgery is performed and provides the surgeon with essential useful feedback on the cognitive status of patients [14].

Nevertheless, many patients report experiencing anxiety during awake craniotomy. Previous studies have evaluated the effects of music on patient anxiety during any awake medical procedures, such as nasal bone fracture reduction [15], parturition [16], transrectal prostate [17] and breast [18] biopsy, extracorporeal shock wave lithotripsy [19], colonoscopies [20], dental extractions [21], carotid endarterectomy [22], and dialysis catheter implantation [23], as well as pain and blood pressure improvement [1,6,24]. Regarding awake craniotomy, a study reported that providing music listening when patients were in the waiting room and during surgery reduced anxiety and reached the goal of improved human and perioperative care [25]. This study is supported by a previous qualitative study which reported that the effects of listening to major- and minor-key musical pieces on patients undergoing awake craniotomy could help in the design of interventions to alleviate anxiety, stress, and tension [26].

In this case report, an RTNT (preceded and followed by music listening) for resections in the left temporal–parietal lobe was performed with the following specific aims:

(1) To show a complete view of the cognitive functions of the patients and to verify how the neuropsychological status evolves during resection;
(2) To test the hypothesis that listening to music during AS decreases the patient’s anxiety and agitation.

## 2. Materials and Methods

The present case report was conducted according to the Declaration of Helsinki, the Guidelines for Good Clinical Practice, and the Strengthening the Reporting of Observational Studies in Epidemiology (STROBE) guidelines [27], and it was approved by the local ethics committee for human experimentation (Prot. N. 1668/01DG).

### 2.1. Pre and Post-Operative Neuropsychological Evaluation

Neuropsychological assessment has been run at baseline (2–3 days before surgery), discharge from the hospital (2–3 days after surgery), and follow-up (1 month and 3 months). Cognitive status has been assessed using the Mini Mental State Examination (MMSE) [28], Clock Drawing Test (CDT) [29], Frontal Assessment Battery (FAB) [30], Babcock Story Recall Test (BSRT) [31], Digit Span Forward and Backward (DS-F, DS-B) [32], Attentional Matrices (AM) [33], Verbal Fluency for letters (VF-L) and for categories (VF-C) [34], Boston Naming Test (BNT) [35], Trail Making Test (TMT) parts A and B [36], Screening Test for Ideo-Motor Apraxia (STIMA) [37], Oral Apraxia (OA) [38], and Copying of Geometric Figures (CGF) [39].

The presence/absence of neuropsychiatric symptoms was evaluated with the Neuropsychiatric Inventory (NPI) [40].

### 2.2. Operative Setting and Procedures

The patient underwent surgery lying in the supine position with his left shoulder uplifted by a pillow. The right arm was placed laterally horizontally on a special armrest. The left arm was free and, to make the position more comfortable, resting on a pillow that the patient held to his chest. The head was only tilted to the right, and not raised or hyperextended, and held in place with a Mayfield–Kees head holder. This last procedure was adjusted with the patient awake to increase patient comfort; we slowly agreed with the patient and the head was angled to the left by about 60 degrees. Sterile drapes were positioned to allow access to the patient’s face for anesthetists and the psychologist, in order to receive and respond to commands during cognitive testing.

Regarding the anesthetic management strategy for AS, the following steps were carried out:

(1) In the preoperative phase, intramuscular clonidine is administered in the evening before surgery and in the morning half an hour before, at a dosage of 2 µg/kg in order to obtain the right anxiolysis;
(2) On the day of the surgery, in the first phase, blocks of the nerves of the scalp are performed with local anesthesia to avoid not only pain during the surgical cut but above all the distress during the placement and removal of the cranial blocker, which certainly involves strong bone tension [41];
(3) The chosen strategic option for awake craniotomy has been MAC (monitored anesthesia care), which involves analgo-sedation via administering Dexmetomidine and Remifentanil in continuous intravenous infusion, allowing the patient to be sedated and in comfort, but contactable and spontaneously breathing [42].

**Figure 1:**
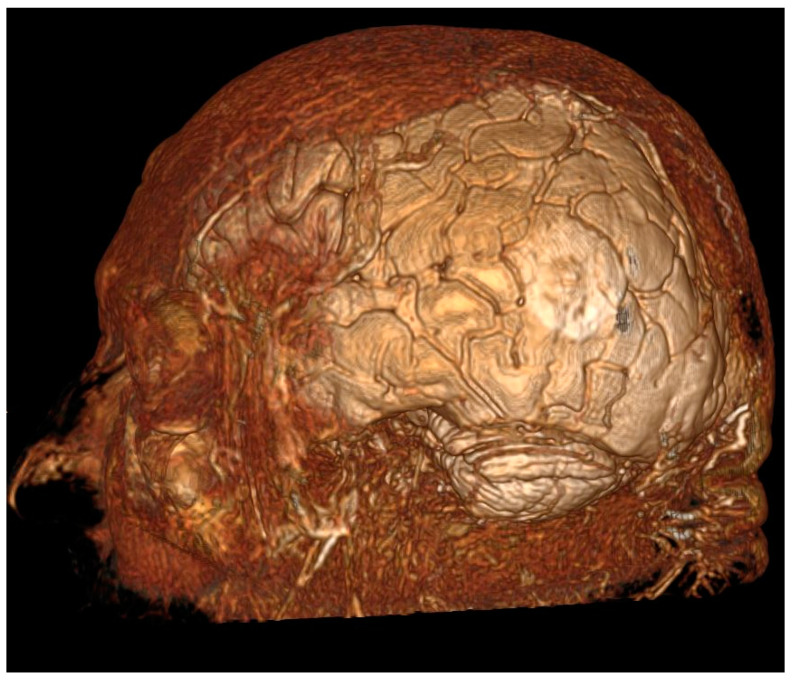
3D reconstruction showing (white area) the localization of the tumor.

After a wide ∩-shaped incision in the left temporo-parieto-occipital region, a 6.5 × 6.5 cm craniotomy was performed. The craniotomy shape was conducted under neuronavigation guidance in order to perform mapping in areas adjacent to the lesion as well [43,44]. The neuro-navigator defined the cortical edges of lesions and established the site of the corticectomy and the trajectory in the approach to subcortical lesions; the corticectomy in our case was performed in an area between the left angularis gyrus and left supramargina, to gain access to the deep temporo-parieto-occipital junction (Figure 1). Before removing tumor or tumor-infiltrated brain tissue, it was remembered that neurological functions can also be found in the same areas [45,46,47,48].

### 2.3. RTNT

The criteria we used to perform RTNT were the same as those used for AS [49]. RTNT was started at the beginning of the resection and ended at the beginning of hemostasis.

The battery of tasks included in RTNT was selected from published neuropsychological batteries available in Italian normative data. Tasks encompassing a wide range of cognitive functions have been included to have an exhaustive intraoperative neuropsychological battery. From the extensive list of tasks, a neuropsychologist selected a series of tasks according to lesion localization, magnetic resonance imaging (MRI) results, and the preoperative neuropsychological profile. The task sequence follows a fixed order with regard to an area. The sequence of tasks was repeated (presenting a different stimuli list for each sequence) until the end of the resection. In each test for a patient, the items were presented for about 30 s for each task, in a rotating manner. In this way task, assessment and task switching served as quick and dynamic methods for immediate dysfunction detection. As soon as the patient exhibited a decrement, the neurosurgeon was immediately informed and carried on with the surgical technique already described.

When this sequence was completed and if patient performance was within the normal range, we restarted testing using the first task and followed the same sequence but with different items. On the contrary, if a patient showed a decrement, we performed in-depth testing.

The following tests were performed when the patient arrived in the operating room and before starting the anesthetic approach (T0) and during tumor excision (T1 and T2): DS-F, DS-B, VF, BNT, and sensory-motor profile awake (SMP-A) [50].

### 2.4. Music Listening

At T0, T1, and T2 (before performing the neuropsychological tests), music listening was applied: a series of songs were chosen by the patient and followed a sensitivity linked to a music therapy approach.

The chronology of the songs was therefore studied, trying to create a sort of emotional path led by the musical melodies and songs that were part of the patient’s youthful experience.

The rhythms, melodic characteristics, and the concepts expressed through the melodies and the lyrics of the songs have been taken into account.

## 3. Case Report

### 3.1. Patient Information

The present report describes the case of the second patient FS, a 66-year-old right-handed man with 8 years of education. He is a shopkeeper. His personal and medical history did not report comorbidities before the diagnosis of the tumor. No family history of epilepsy or other neurological diseases was reported.

For some months, he has been reporting alterations in expressive language and memory deficit, with more evident worsening in the last two weeks.

**Figure 2:**
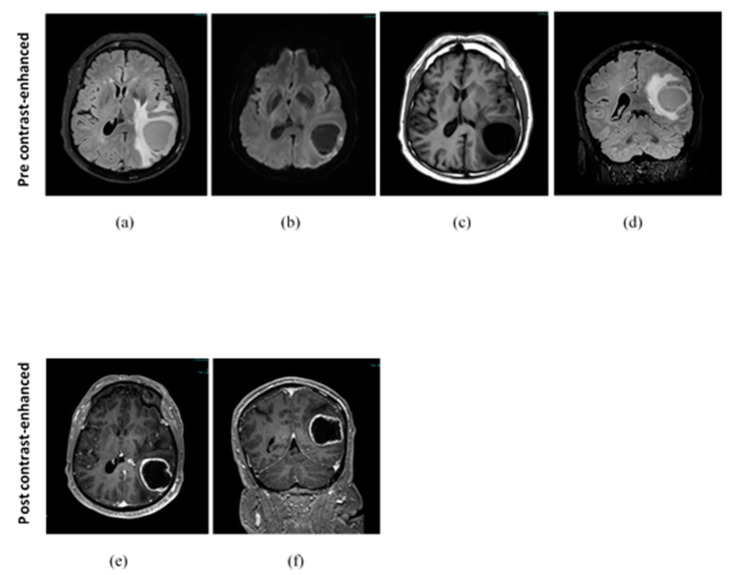
T1-weighted, Flair MRI images and spectroscopy acquired pre-surgery. (**a**) Pre-contrast-enhanced Flair MRI axial slice; (**b**) pre-contrast-enhanced diffusion-weighted imaging at b1000 axial slice; (**c**) pre-contrast-enhanced T1-weighted MRI axial slices; (**d**) pre-contrast-enhanced Flair MRI coronal slice; (**e**) post-contrast-enhanced T1-weighted MRI axial slices; (**f**) post-contrast-enhanced T1-weighted MRI coronal slices.

The patient was examined with MRI, which evidenced a lesion in the left temporo-occipital–parietal cortex of likely heteroplastic nature; the lesion (46 mm measured on the MRI image) was characterized by abundant central necrosis and a solid component with intense marginal contrast enhancement (CE), which corresponded to a significant increase in choline and the presence of lipids (Figure 2). The hypothesis of an awake surgery was considered. His neurological assessment was unremarkable regarding the sensorium, cranial nerves, motor, sensory, cerebellar, gait, reflexes, meningeal irritation, and long tract signs; only cognitive aspect results are worthy of further study using psychometric scales.

Blood samples, including routine blood count, kidney and liver function test, serum lipids, glucose level, serum lactate, lactic acid dehydrogenase, serum immunoglobulin, thyroid hormones and autoantibodies (anti-TPO), and routine autoimmunity testing (ANA, ENA, ANCA, and anti-phospholipids antibodies), were all normal.

### 3.2. Clinical Findings

At baseline (Table 1), the patient showed (1) a severe impairment of working memory (DS-B = 0.25), long-term memory, shifting ability (TMT-B = 482.00), and verbal fluency (VF-L = 5.00; VF-C = 15.00); (2) a mild–moderate deficit of selective attention (AM = 44.25); executive and visuospatial functions (FAB = 10.00; CDT = 3.00); lexical naming performance (BNT = 41.00); bucco-facial, ideomotor and constructive praxia (OA = 8.00; STIMA = 8.00; CGF = 9.75); (3) anxious–depressive symptoms (NPI = 17.00).

### 3.3. Timeline and Intra-Operative Evaluations

As shown in Table 2, at T0, before AS and after music listening, the patient reported a decrease in depression and anxiety (NPI = 10.00).

During AS, improvement was shown in all parameters collected, respectively, at T0, T1, and T2: DS-F (5.25 vs. 6.25 vs. 6.25), DS-B (0.25 vs. 1.25 vs. 3.25), VF-L (5.00 vs. 10.00 vs. 14.00), VF-C (15.00 vs. 24.00 vs. 32.00), and BNT (44.00 vs. 50.00 vs. 55.00). No changes turned up on the SMP-A (100.00 vs. 100.00 vs. 100.00) scale.

After excision and music listening (T2), the patient reported a further decrease in depression and anxiety (NPI = 7.00).

After the resection, histological exams confirmed the neuro-radiological suspicion of GBM.

### 3.4. Follow-Up and Outcomes

As shown in Table 3, at three days post surgery and at follow-up appointments after one month and three months, the patient reported only an isolated working memory (DS-B = 3.25 vs. 4.25 vs. 3.25) and praxic–constructive capacity (CGF = 10.75 vs. 10.75 vs. 11.75) impairment, the absence of depression, and a reduction in anxiety symptoms (NPI = 6.00 vs. 4.00 vs. 4.00).

**Figure 3:**
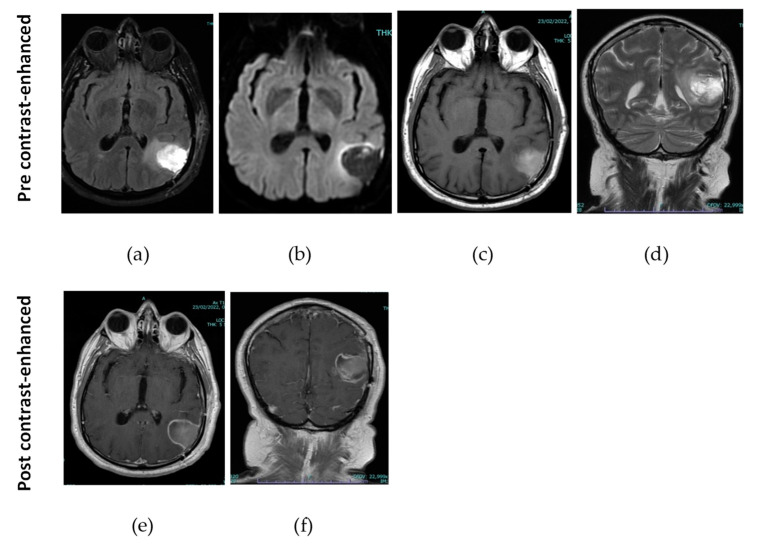
T1, T2-weighted, Flair MRI images acquired after a month of follow up. (**a**) Pre-contrast-enhanced Flair MRI axial slice; (**b**) pre-contrast-enhanced diffusion-weighted imaging at b1000 axial slice; (**c**) pre-contrast-enhanced T1-weighted MRI axial slices; (**d**) pre-contrast-enhanced T2-weighted coronal slice; (**e**) post-contrast-enhanced T1-weighted MRI axial slices; (**f**) post-contrast-enhanced T1-weighted MRI coronal slices.

After a month of follow up, the MRI images (Figure 3) reported the results of left temporo-parietal craniotomy surgery for the removal of GBM with an inhomogeneous, partly hematic surgical cavity, delimited by an irregular enhancement border after the administration of CE in some points with nodular characteristics.

## 4. Discussion

The study of cortical and cortico-axonal connectivity represents the new frontier of cognitive neuroscience for understanding the evolution of thought and mind. Furthermore, preserving cortical and axonal connectivity is the goal of brain tumor surgery in order to avoid the onset of permanent post-operative neuropsychological deficits. The surgeon can be trained to sew the surgical trajectory on the particular patient through interrupting or dislocating the fibers, minimizing injury to the neighboring fibers. White fibers can exhibit a variety of modifications, as also mentioned by Duffau [51], including morphological distortion brought on by a mass effect, tumor cell infiltration, the presence of edema, complete interruption, and occasionally functional reorganization. High-grade gliomas, on the other hand, can invade white matter tracts, leading to the displacement, rupture, and subsequent modification of the white matter signal [52]. White matter, in contrast to the cerebral cortex, shows relatively limited functional remodeling; hence, sparing fiber bundles is crucial [53].

Consequently, the preoperative, intraoperative, and postoperative neuropsychological evaluation of patients with brain tumors and those who have been treated neurosurgically has become part of clinical protocols. It allows us to evaluate the presence of any neuropsychological deficits and their severity, provides reliable indications regarding the patient’s tolerability of an intervention in AS, and examines the cognitive and emotional motivational status of the patient in the postoperative phase, providing indications of the rehabilitation treatment and the quality-of-life level. It arises according to an individualized clinical–relational process aimed at exploring the interests, tastes, habits, and temperamental and personological characteristics of each patient for an adequate understanding of inter-individual differences. A complete and objective neuropsychological assessment also evaluates the functioning of a wide range of cognitive functions: language, memory, learning, working memory, visuospatial skills, attentional and executive skills, praxic skills, motivation, and emotional and behavioral regulation. Unfortunately, in the neurosurgical field, to date, much attention has been paid to language skills alone, modulating surgical resection techniques and preoperative and intraoperative mapping during awake surgery, to avoid the onset of language and aphasic deficits after surgery. However, very little has been done with respect to other cognitive functions which are equally important for an adequate level of quality of life and are seriously disabling if deficient. Many studies conducted thanks to systematic protocols of neuropsychological assessment highlight, in fact, the constant presence of cognitive problems related to functions other than language in patients suffering from brain tumors or epilepsy who are treated surgically [54,55].

In this case report, it has been shown how fundamental a complete neuropsychological profile of the patient is. The patient not only presented a language deficit but also impairment of working memory; long-term memory; shifting ability; verbal fluency; selective attention; executive and visuospatial functions; and bucco-facial, ideomotor, and constructive praxia; in addition to anxious–depressive symptoms.

Moreover, we demonstrated that before/during AS and after music listening, the patient reported a decrease in depression and anxiety in addition to the improvement of all collected cognitive parameters. These outcomes are in line with other studies that demonstrated the positive effects of music listening on patient satisfaction, anxiety, and depression [56,57]. A study showed that listening to music with headphones obtains relaxing effects comparable to those of midazolam: muscles relax, anxiety vanishes, and stress levels are lowered [58].

Through a neuropsychological evaluation performed at three days post surgery and at follow-up appointments after one month and three months, it has been possible to report the further cognitive and affective improvements of the patient. Another important factor to be taken into consideration during the neuropsychological assessment and the preoperative and intraoperative mapping of the cognitive and neurological functioning of patients with GBM is the possibility—almost systematic, as clinical practice suggests—that the brain has undergone of the reorganization of functional networks through processes of brain plasticity. This, among other things, would explain the heterogeneity of cognitive and neurological symptoms among GBM patients affecting the same brain areas.

Brain plasticity could be defined as a continuous process of remodeling and reorganizing neuronal synapses in the short, medium, and long term; during phylogenetic and ontogenetic development; and in the presence of brain lesions. It strengthens even more the modern neuroscientific conception of the brain as a complex and dynamic organ, not fixed, and emphasizes the appropriateness of the functional and neuropsychological neuro-oncological approach towards patients with GBM. The cognitive and behavioral consequences of the phenomenon of neuronal plasticity in these patients have been extensively studied [56] and justify the inhomogeneity of the preoperative and intraoperative data obtained from neurocognitive evaluations and from mapping methods. In other words, it should not be a surprise if, for example, the preoperative mapping of the linguistic functions of a patient affected by GBM who does not present linguistic deficits upon neurocognitive evaluation yields results that affect areas of the cortex not properly held responsible for linguistic and distant functions, or even contralateral to the injury. The phenomenon of neuronal plasticity is also almost systematic in patients suffering from gliomas, compared, for example, to patients affected by stroke, due to the very nature of the disease, which develops over time; over time, the glioma grows at the same time as the brain grows [59]. Cortico-subcortical reorganization can also be observed and confirmed during mapping via intraoperative stimulation induced by the presence of a brain tumor.

Plastic reorganization mechanisms are also observable in the postoperative phase [60]. Therefore, the observation of the phenomenon of the reorganization of cortical circuits through the various mapping methods and the standardized neuropsychological evaluation in neurosurgery involves different fundamental therapeutic implications for achieving the objectives that the functional neuro-oncological approach aims to achieve. It allows the surgical treatment to be extended to eloquent or near-eloquent areas, maximizes the surgical resection of the lesion, and minimizes the risks of post-operative neuropsychological and neurological sequelae through improving the quality of life of patients [61].

As usually happens, the limitations of the case report lie in the impossibility of drawing generalizations, establishing cause–effect relationships, and the danger of over-interpretation. In particular, in this case report, a control is not described; for this reason, the ability to draw conclusions is severely limited.

Cooperation with other clinicians and the use of other research methodologies are needed. An investigation of current neuropsychological approaches and working towards agreed and standardized protocols could be prospects for future research.

## 5. Conclusions

In conclusion, in light of what has been argued up to now, the brain of every human being is organized differently from all the others in normal conditions. This is supported by the most recent scientific research in neuroscience that suggests the existence of inter-individual differences in the organization of neuronal networks at the cortical and subcortical levels between one brain and another, significantly beyond the classic conceptions of the localization of human cognition and emotion, opening the doors to dynamic and complex approaches and models. The brains of GBM patients also undergo functional neuroplasticity phenomena that make them even more different and complex than the norm. The surgical treatment of such patients, therefore, is plausible if one uses, for the modulation of resection techniques, the data coming from the neuropsychological evaluation and from the mapping methods that do not disregard the singularity of the brain and of the patient’s personality.

## Figures and Tables

**Table 1:** Patient’s psycho-behavioral aspects and neuropsychological performance at baseline.

	Score	Remark
Mini Mental State Examination (MMSE)	19.53	Mild cognitive impairment
Neuropsychiatric Inventory (NPI)	17	Depression, Anxiety, Insomnia
Clock Drawing Test (CDT)	3	Mild to moderate visuo-spatial disorganization
FrontalAssessmentBattery (FAB)	10	Impaired executive functions
Trail Making Test (TMT)-A	47	Mild impairment
Trail Making Test (TMT)-B	283	Severe impairment
Matrici Attentive (MA)	44.25	Mildimpairment
DigitSpan–Forward (DS-F)	6.25	No compromised
DigitSpan–Backward (DS-B)	0.25	Severe impairment
Babcock Story Recall Test (BSRT)	3.3	Severe impairment
Verbal Fluency for letter (VF-L)	5	Severe impairment
Verbal Fluency for category (VF-C)	15	Severe impairment
Boston Naming Test (BNT)	41	Mild to moderate impairment
Copying of Geometric Figures (CGF)	9.75	Moderate impairment
Screening Test for Ideo-Motor Apraxia (STIMA)	8/10	Mild impairment
OralApraxia (OA)	8/10	Mild impairment

**Table 2:** Patient’s psycho-behavioral aspects and neuropsychological performance at different testing times: before starting the anesthetic approach (T0) and during tumor excision (T1 and T2).

Test	T0	T1	T2
Time	08:45	11:00	12:23
Neuropsychiatric Inventory (NPI)	10	10	7
DigitSpan–Forward (DS-F)	5.25	6.25	6.25
DigitSpan–Backward (DS-B)	0.25	1.25	3.25
Verbal Fluency for letter (VF-L)	5	10	14
Verbal Fluency for category (VF-C)	15	24	32
Boston Naming Test (BNT)	44	50	55
Sensory-motorprofileawake (SMP-A)	100	100	100

**Table 3:** Patient’s psycho-behavioral aspects and neuropsychological performance at three days post surgery and follow-up of one month and three months.

Test	3 Day Post-Surgery Score	1 Month-Follow Up Score	3 Month-Follow Up Score
Mini Mental State Examination (MMSE)	25.53	28.53	28.53
Neuropsychiatric Inventory (NPI)	0	4	4
Clock Drawing Test (CDT)	2	1	1
FrontalAssessmentBattery (FAB)	11	15	16
Trail Making Test (TMT)-A	46	32	30
Trail Making Test (TMT)-B	107	31	105
Matrici Attentive (MA)	44.25	52.25	49.25
DigitSpan–Forward (DS-F)	5.25	6.25	5.25
DigitSpan–Backward (DS-B)	3.25	4.25	3.25
Babcock Story Recall Test (BSRT)	4.3	15.7	12.6
Verbal Fluency for letter (VF-L)	14	17	18
Verbal Fluency for category (VF-C)	40	42	39
Boston Naming Test (BNT)	56	59	53
Copying of Geometric Figures (CGF)	10.75	10.75	11.75
Screening Test for Ideo-Motor Apraxia (STIMA)	10/10	10/10	10/10
OralApraxia (OA)	10/10	10/10	10/10

## Data Availability

Not applicable.
